# Mathematical epidemiologic and simulation modelling of first wave COVID-19 in Malaysia

**DOI:** 10.1038/s41598-021-99541-0

**Published:** 2021-10-20

**Authors:** Muhammad Rezal Kamel Ariffin, Kathiresan Gopal, Isthrinayagy Krishnarajah, Iszuanie Syafidza Che Ilias, Mohd Bakri Adam, Jayanthi Arasan, Nur Haizum Abd Rahman, Nur Sumirah Mohd Dom, Noraishah Mohammad Sham

**Affiliations:** 1 Institute for Mathematical Research, Universiti Putra Malaysia, 43400 UPM Serdang, Selangor Malaysia; 2 Department of Mathematics and Statistics, Faculty of Science, Universiti Putra Malaysia, 43400 UPM Serdang, Selangor Malaysia; 3 Regent International School, Wisma Lourdes, 41000 Klang, Malaysia; 4 Environmental Health Research Centre, Institute for Medical Research, 40170 Shah Alam, Selangor Malaysia

**Keywords:** Computational biology and bioinformatics, Diseases, Mathematics and computing

## Abstract

Since the first coronavirus disease 2019 (COVID-19) outbreak appeared in Wuhan, mainland China on December 31, 2019, the geographical spread of the epidemic was swift. Malaysia is one of the countries that were hit substantially by the outbreak, particularly in the second wave. This study aims to simulate the infectious trend and trajectory of COVID-19 to understand the severity of the disease and determine the approximate number of days required for the trend to decline. The number of confirmed positive infectious cases [as reported by Ministry of Health, Malaysia (MOH)] were used from January 25, 2020 to March 31, 2020. This study simulated the infectious count for the same duration to assess the predictive capability of the Susceptible-Infectious-Recovered (SIR) model. The same model was used to project the simulation trajectory of confirmed positive infectious cases for 80 days from the beginning of the outbreak and extended the trajectory for another 30 days to obtain an overall picture of the severity of the disease in Malaysia. The transmission rate, β also been utilized to predict the cumulative number of infectious individuals. Using the SIR model, the simulated infectious cases count obtained was not far from the actual count. The simulated trend was able to mimic the actual count and capture the actual spikes approximately. The infectious trajectory simulation for 80 days and the extended trajectory for 110 days depicts that the inclining trend has peaked and ended and will decline towards late April 2020. Furthermore, the predicted cumulative number of infectious individuals tallies with the preparations undertaken by the MOH. The simulation indicates the severity of COVID-19 disease in Malaysia, suggesting a peak of infectiousness in mid-March 2020 and a probable decline in late April 2020. Overall, the study findings indicate that outbreak control measures such as the Movement Control Order (MCO), social distancing and increased hygienic awareness is needed to control the transmission of the outbreak in Malaysia.

## Introduction

The first identified case of coronavirus disease 2019 (COVID-19) was reported in mainland China, in the city of Wuhan on December 31, 2019^1^. Ever since then, the geographical spread of the epidemic was swift and beyond control, hence turning it into a pandemic^2^. Beginning on February 26, 2020, the number of COVID-19 cases increased rapidly worldwide compared to inside China. On January 30, 2020, the World Health Organization (WHO) declared the outbreak a Public Health Emergency of International Concern^3^. On March 11, 2020, it was said a pandemic for almost every part of the world^4^. COVID-19 is caused by the severe acute respiratory syndrome coronavirus 2 (SARS-CoV-2), resulting in more than 4,500,000 deaths from January 2020 until September 2021^5^. The death toll of COVID-19 is hugely more extraordinary than that of the other infectious diseases caused by viruses such as SARS, MERS, Ebola, and H1N1^6^. At the onset of COVID-19, patients usually show symptoms connected to viral pneumonia, fever, cough, sore throat, myalgia, and fatigue^7–12^. The incubation period of COVID-19 is generally between two to 14 days or longer and usually takes 5 days as the average^13,14^. The virus can spread from an individual to another through respiratory droplets and close contact^15^.

**Figure 1:**
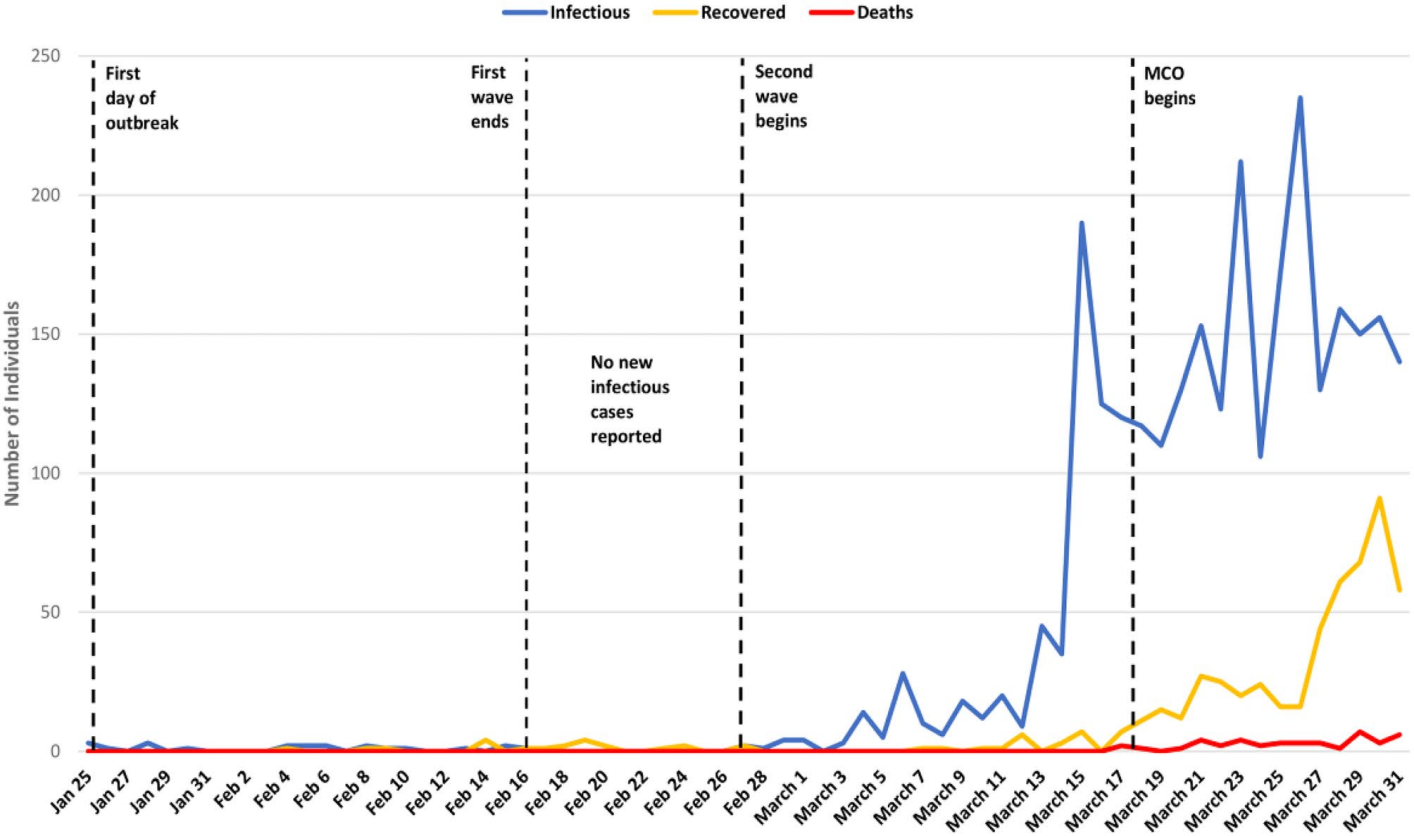
Malaysian scenario of COVID-19 outbreak.

Malaysia experienced the first incidence reported on January 25, 2020, through three imported cases of COVID-19 involving three tourists from China who had entered Malaysia via Johor from Singapore on January 23, 2020. The first wave of the outbreak commenced on the first incidence day until February 16, 2020. During these 23 days, only 22 confirmed cases were recorded with eight recovered patients and zero deaths, with minimal outbreak control measures exercised. Moreover, there were no new cases recorded for the following 10 days. However, on February 27, 2020, a recent incident involving two cases marked the beginning of the second outbreak wave in Malaysia. The first 18 days of the second wave did not see a tremendous increase in the number of new cases; however, on March 15, 2020, the numbers had a sudden rise to 190 individuals from only 35 on the previous day. This alarming rate was due to identifying large clusters of susceptible individuals in contact with infectious individuals (s). Following that, the first death incidence involving two cases occurred on March 17, 2020. The sudden hike and severe outbreak spread changed the Malaysian context of the epidemic from being in control to a dangerous state. They triggered the Movement Control Order (MCO) implementation on March 18, 2020^16^. Since the first rise on March 15, 2020, the inclining trend of new cases each day continued with an average of 150 cases per day and was hit with a major spike of 235 cases on March 26, 2020. On top of that, the number of deaths started spiraling^9^ upwards, with more than 60% of fatalities were patients over the age of 60 years, which included those with underlying conditions such as hypertension and diabetes (as of March 31, 2020), in line with the findings from Guan et al.^10^ clinical progress study. The Malaysian scenario of COVID-19 outbreak from January 25, 2020 to March 31, 2020 can be visualized in Fig. 1.

A well-known contradiction during the early pandemic is that only minimal knowledge is available about the disease; however, the need for extensive such information is extremely high^17^. This contradiction is evidently true for the case of COVID-19. As such, research on modelling using available data plays a crucial role in the times of a pandemic^18,19^. Accordingly, estimation of infectious count over time can provide a better understanding of the current epidemiological situation in many countries including Malaysia. It can provide insights into the measurable effect of undertaken outbreak control measures^20^. Analysis providing such estimations enables predictions of potential future growth to assist in risk estimation of regional countries and planning alternative interventions or increasing the intensity of existing interventions^20–22^. Concerning the perturbing situation in Malaysia, assessing the infectious trend of COVID-19 is crucial to measure the pandemic's severity, as evidenced by the enormous growth from three to 2766 cases within only 67 days (March 31, 2020).

Nevertheless, performing such analyses, especially in real time is often difficult due to constraints such as delay in symptom appearance resulting from the incubation period and delay in confirmation of positive cases resulting from limitations in testing and detection capacity^20,23^. Mathematical modelling of infectious diseases can help overcome the constraints caused by the delays and uncertainty^24^. The most common modelling approach to simulate a contagious disease outbreak's probable outbreak trajectory and severity is the Susceptible-Infectious-Recovered (SIR) model^9^. As anticipated, several studies have widely applied the SIR model^25,26^ and its extensions, such as the Susceptible-Exposed-Infectious-Recovered (SEIR) model^27–32^, the Susceptible-Exposed-Infectious-Hospitalized-Recovered (SEIHR)^29^ and the Susceptible (S), Exposed/pre asymptomatic (E), Asymptomatic (A), Symptomatic (I), Recovery (R) and the Virus in the Environment, thus, on surfaces (V)^33^ to the current COVID-19 outbreak at global and national levels. Sun and Weng^34^ constructed an adjusted model with two novel features: the asymptomatic population and recovery threshold behaviour. Meanwhile, auto-regressive integrated moving average (ARIMA) models were created by Ceylan^35^ to estimate the epidemiological trend of COVID-19 prevalence in Italy, Spain, and France, Europe's most impacted countries. Giordano et al.^36^ modified the SIR model to include susceptible (S), infected (I), diagnosed (D), ailing (A), recognized (R), threatened (T), healed (H), and extinct (E) in distinguishing between infected individuals based on whether or not they have been diagnosed and the severity of their symptoms. Then, in 2021, Asomoah et al.^37^ extended the generalized SEIR epidemic model to include a Susceptible (S), Exposed (E), Asymptomatic (A), Quarantined asymptomatic (Q), Severely infected (I_s_), Hospitalized (H), Recovered (R), Recovered asymptomatic (R^A^), Deceased (D), and Protective susceptible (S_p_) compartmental structure to describe COVID-19 dynamics. Other, Yamamoto et al.^38^ provided a spatio-temporal approach for quantifying regional compliance with the US COVID-19 mitigation strategies. In Malaysia, several studies have been made by researchers such as Wong et al.^39^, who modified the SIR model under vaccine intervention in several localities of Malaysia by using the simulation of the COVID-19 spread. Salman et al.^40^ use a simple universality class of the SIR system and adaptations thereof to undertake scenario analysis for COVID-19 in Malaysia (i.e., the inclusion of temporary immunity through the reinfection problems and limited medical resources scenarios leads to the SIRS-type model). Furthermore, Abidemi et al.^41^ developed a deterministic compartmental model to examine the impact of various pharmaceutical and nonpharmaceutical control measures on COVID-19 population dynamics in Malaysia.

Using the SIR model, we simulated the infectious trend of COVID-19 in Malaysia to estimate the COVID-19 transmission pattern for a period of 67 days. The simulation is used to obtain an overall picture of COVID-19's potential severity in Malaysia. We searched Google Scholar, medRxiv, arXiv for peer-reviewed articles, preprints, and research reports on the modelling of coronavirus disease 2019 (COVID-19) severity using the search terms "COVID-19 modelling", "epidemic model COVID-19", "mathematical modelling COVID-19", "SIR COVID-19" and "SEIR COVID-19" up to March 30, 2020. No language restrictions were applied. We identified 12 papers that were relevant in the context of mathematical modelling for COVID-19 that can be applied to the Malaysian scenario. Most of the papers focused on the outbreak at the global level, in Hubei, China, China in general, South Korea, Italy, and Iran. We also found several estimates of the transmission rates, recovery rates and the basic reproductive numbers used in these papers. Until now, there has been scarce information in understanding the changing severity and transmission dynamics of COVID-19 in the Southeast Asia region, particularly Malaysia, which holds the highest number of cases (more than 2500 active cases) at the time of writing (March–April 2020).

In the absence of a complete study for Malaysia or the Southeast Asia region, in particular, we inferred the severity of COVID-19 infectiousness in Malaysia by simulating the infectious count against the actual count. We used the same model to project the simulation trajectory into the future (up to 110 days since day zero of the outbreak) to estimate the approximate number of days for the inclining trend and sudden spikes to decline. Furthermore, we predicted the cumulative number of infectious individuals in order to assess the preparations undertaken by MOH. That is, whether MOH has prepared the minimal number of beds or not. We show that the severity dynamics of COVID-19 in Malaysia is rapidly changing and should be closely monitored. Our findings suggest that outbreak control measures such as stricter enforcement of the Movement Control Order, social distancing and increased hygienic awareness are needed in order to control the local transmission of the outbreak.

## Results

### Actual and simulated current infectious trend

The visualization of the first 67 days of the COVID-19 outbreak in Malaysia is shown in Fig. 1, with the four major timelines marked with a dotted black line. The infectious count is represented by a blue line, the recovered count is represented by a yellow line, and the deaths count is represented by a red line. The SIR model is initialized (at time $t=0$) with the initial conditions $S\left(0\right)={S}_{0}=0.999$ (or $999$ for computational simplicity), and $I\left(0\right)={I}_{0}=0.001$ (or $1$ for computational simplicity), while $R\left(0\right)={R}_{0}=0$. We then obtained the basic reproductive number ${\mathfrak{N}}_{0}$ for this study using the average ${\mathfrak{N}}_{0}$ = 2.44 estimated using stochastic methods in two previous studies^27,31^. It is consistent with the range estimated by WHO^1^ and Soetaert et al.^42^. The average number of days of recovery is assumed to be $D=11$ days based on the first recovered case in Malaysia. It follows that the recovery rate, $\gamma =1/11$ = 0·09. Next, by using ${\mathfrak{N}}_{0}=\beta /\gamma$^43^, we derive the value of the transmission rate, $\beta$ = 2·44(0·09) = 0.22. Finally, with the values of ${S}_{0},{I}_{0},$ and ${R}_{0}$, the differential equations were solved to obtain the values of each compartment at each time point (days) beginning from day zero (January 25 2020) today 67 (March 31, 2020).

**Figure 2:**
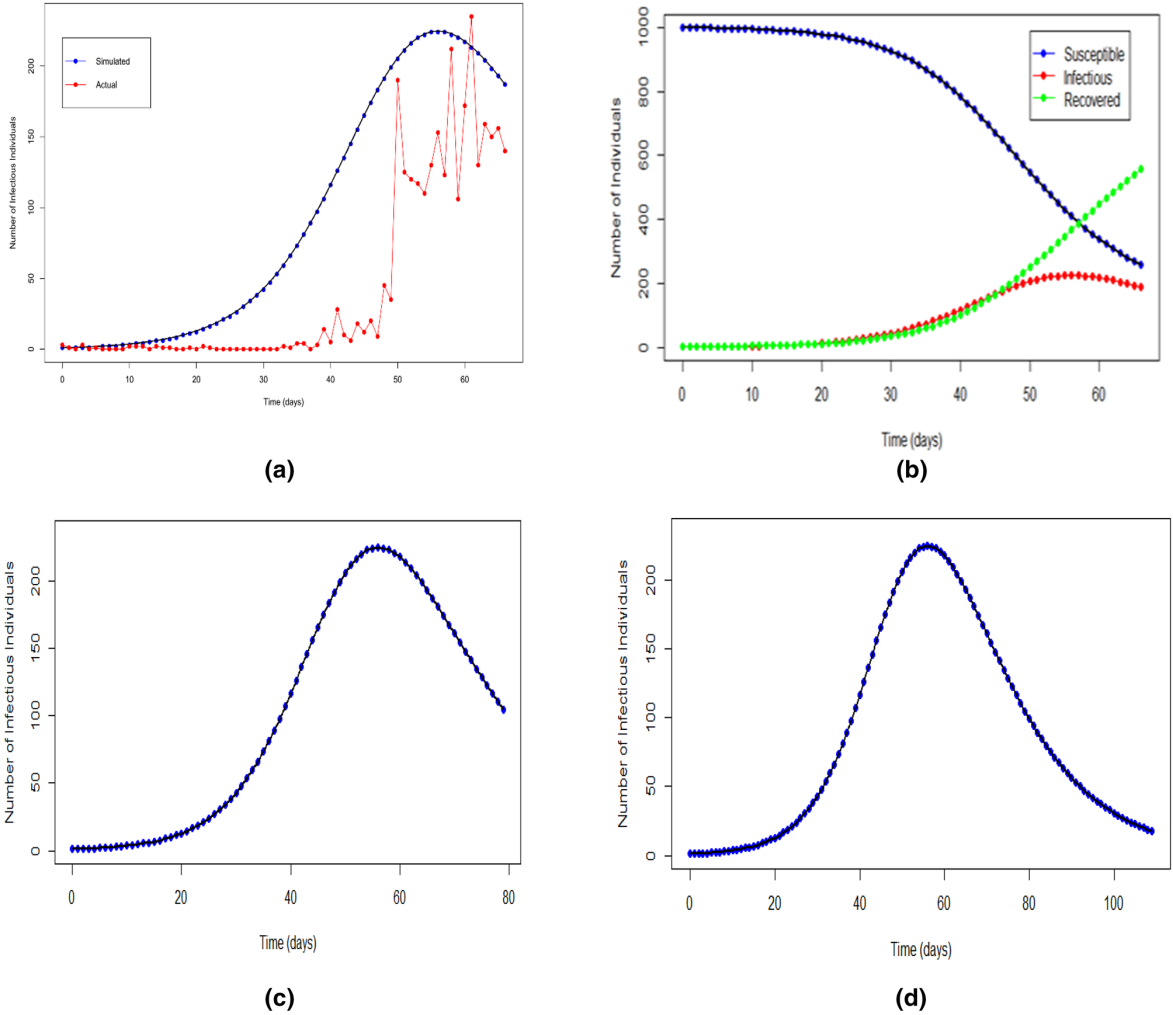
The actual and simulated infectious trend. (**a**) For 67 days, (**b**) simulated SIR compartments, (**c**) simulated projection of 80 days trajectory, (**d**) simulated projection of 110 days trajectory.

It can be seen that in the initial stage (day zero to day 15, Fig. 2a), the simulated counts were approximately close to actual counts. However, after day 20, the simulation had an upwards trend with the peak value of 224 infectious individuals on day 56 (March 21, 2020). The simulation trend started declining after day 58. On the other hand, the actual infectious counts only started increasing drastically after day 50, and there were three major spikes between days 50 and 61. The actual peak value was 235 infectious individuals on day 61 (March 26, 2020).

The standard SIR model was able to approximately mimic the actual trend and predict the actual spikes. The discrepancy in SIR simulation between day 20 to day 50 is due to the nature of the actual counts (discrete values) when there is a sudden spike in the number of confirmed cases. Note that the simulated peak was also approximately close to that of the actual peak. All three simulated compartments of the SIR model for the same period of time are shown in Fig. 2b. The simulated susceptible trend declines after day 30 and further down after day 60. At the same time, the simulated recovered trend inclines after day 30 while the infectious trend was steadily approaching a downward trend. The blue dots represent the simulated number of susceptible individuals, the red dots represent the simulated number of infectious individuals, and the yellow dots represent the simulated number of recovered individuals in the period of 67 days.

### Trajectory projection simulation of COVID-19 in Malaysia

The predictive capability of the SIR model in the previous section is fairly good based on the standard criteria of small quantified discrepancy between the actual and simulated cases. Thus, we used the same initial conditions and parameters' values to simulate the trajectory projection of COVID-19 in Malaysia. Two trajectories were produced: 80 days from day zero (until April 13, 2020) and 110 days from day zero (until May 13, 2020). A number of days for the trajectories were chosen arbitrarily to determine the approximate number of days needed for the infectious trend to completely decline since the first day of COVID-19's establishment in Malaysia.

It can be observed from Fig. 2c that the simulated maximum count is 224 infectious individuals in a day. Thereafter, the simulated line dropped to below 200 infectious individuals in a day and further to below 110 infectious individuals at the end of the trajectory on April 13, 2020. For the second trajectory (Fig. 2d), which is the extended trajectory of 30 days from the previous one, the infectious count trend declines steadily by exhibiting a downward trend from day 80 (April 13, 2020) and reaches the lowest point with less than 20 cases in a day on May 13, 2020, which indicates that the severity of COVID-19 in Malaysia may reduce by mid-May, 2020. The actual infectious count reported on April 13, 2020, was 134 new infectious individuals. In between April 14 until April 23, 2020, actual data showed a downward trend. The actual infectious counts reported were 170, 85, 110, 69, 54, 84, 36, 57, 50 and 71 new infectious individuals respectively within that time period. Except for the spike on April 14, 2020, the preceding counts maintained below the generated SIR curve. As such, evidence from actual data on infectious individuals has a strong correlation to the fact that our SIR curve is based on $\beta$ = 0·22. This implies a strong assumption that infection from an infectious individual to a susceptible individual in Malaysia is at a conservative rate. That is, at the high end, an infectious individual in Malaysia will transmit the disease to a susceptible individual every 4 days, while at the low end, in 5 days.

**Figure 3:**
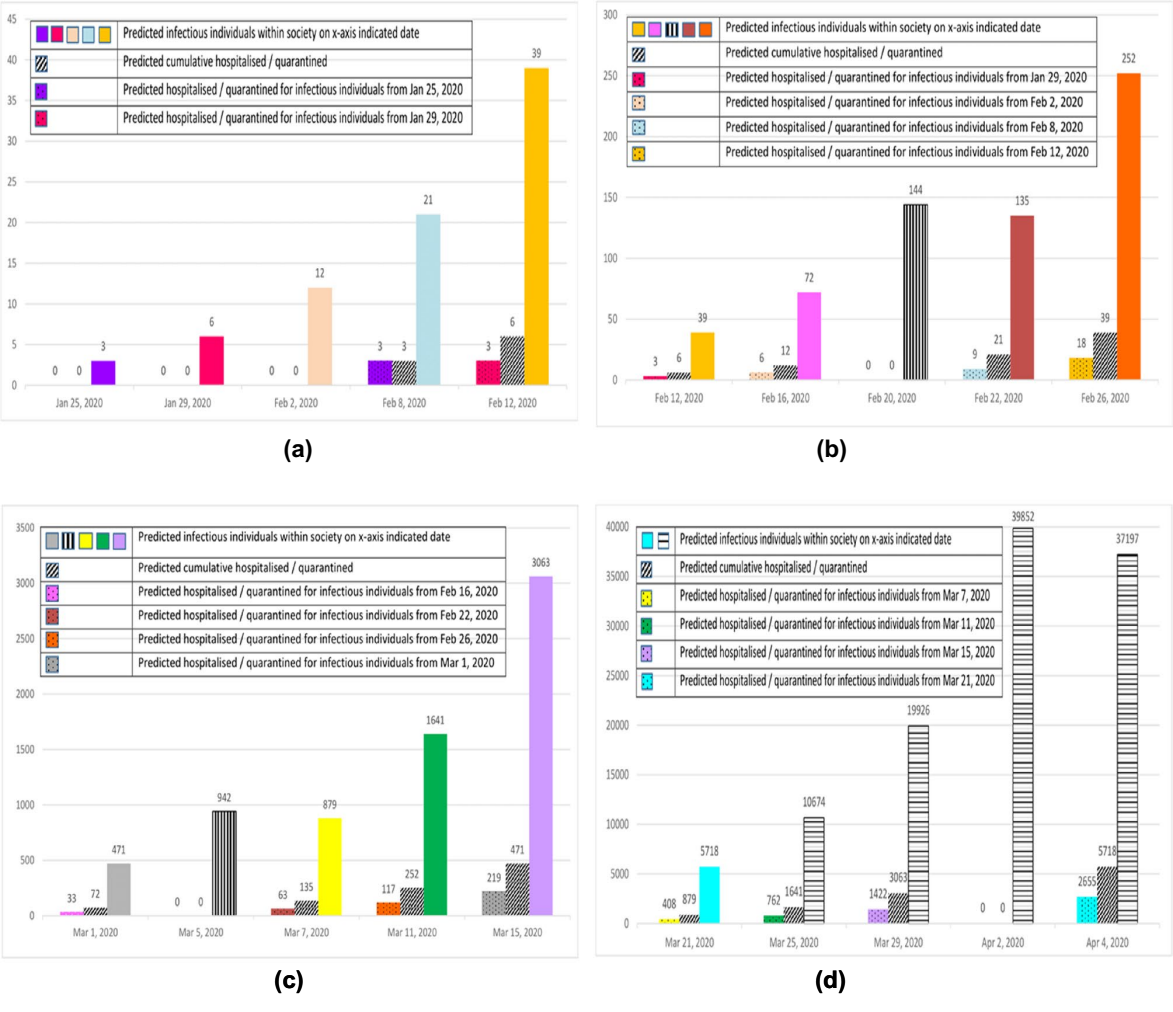
Predicted Cumulative according to 14-day period. (**a**) Jan 25, 2020 and Feb 12, 2020. (**b**) Feb 12, 2020 and Feb 26, 2020. (**c**) March 1, 2020 and March 15, 2020. (**d**) March 21, 2020 and April 4, 2020. The colour codes in Fig. 3a–d were used to identify the 14-day period of each infectious cohort beginning from the first day the cohort becomes infectious. For example, in Fig. 3a the colour purple-blue for the first cohort of infectious individuals on Jan 25, 2020: it is predicted that there are three infectious individuals within the society, where three of them will show symptoms and be hospitalised after a 14-day period on Feb 8, 2020.

Figure 3 present an account of our predicted values when compared to actual values reported by the MOH^45^ Malaysia on a daily basis. The red dots represent the predicted cumulative infectious cases, and the blue dots represent the actual cumulative infectious cases. The data points of the actual infectious cases from March 15, 2020, to March 31, 2020, are provided on the plot to depict how close the predictions are. The predicted cumulative infectious cases with ${I}_{0}$ = three on 4 days intervals are shown in blue bars, while the actual cumulative infectious cases are shown in orange bars.

**Figure 4:**
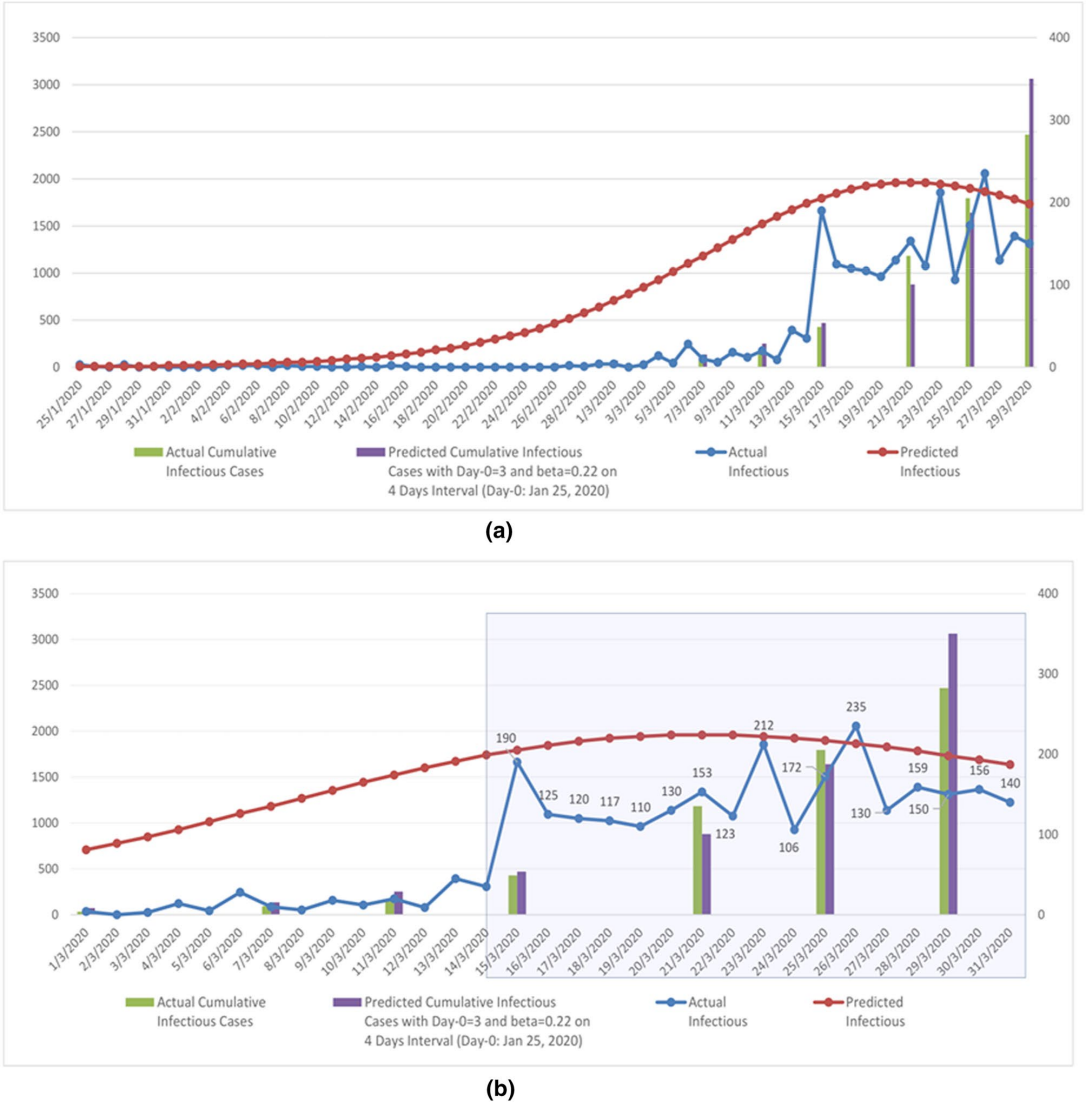
Actual versus Predicted Cases by date; (**a**) Jan 25 to Mar 31, 2020. (**b**) Mar 1 to Mar 31, 2020.

Summarizing Fig. 3 indicates that on average the difference between actual and simulated cases is about 10% (for cumulative cases) and about (for daily cases). Hence, the SIR model produces predicted daily values that are not far off from the actual daily values. As such, the SIR transmission rate, $\beta$ = 0·22, which corresponds to a hypothesized scenario whereby an individual will infect another individual within a 4-day interval, should not be taken lightly. Furthermore, a one-to-one transmission on an interval of 4 days can be seen to be rather conservative. Nevertheless, as shown in Fig. 4, even at this rate, the exponential growth rate is visible.

## Discussions

Based on the SIR model's simulation, we extend our discussion to predict cumulative positive infectious cases of COVID-19 in Malaysia. This discussion is subject to further analysis since it is not conclusive in nature. However, we would like to highlight at this point of our argument that discussions provided here are based on definitions within the literature. We will base our discussion on $\beta$ = 0·22, which implies a one-to-one transmission after 4 days. This assumption is conservative.

We hypothesize that after an individual becomes infected, thus becoming an infectious individual, he will only display symptom(s) after 14 days in order to be confirmed positive with the COVID-19 disease and will be hospitalized thereafter. As such, after being hospitalized, he will not be able to be in contact with other susceptible individuals. This hypothesis is well supported by several studies that include Cruz et al.^46^, Del Rio and Malani^47^, Han and Yang^48^, and Walsh et al.^49^. Furthermore, we hypothesize that during the 14 days period, an infectious individual will be able to spread the disease to another individual in a 4-day interval. This hypothesis is supported by factors such as living conditions, social movements, exposure within known clusters, climate, and environmental conditions^12,13,50–53^.

We assume that the first three infectious individuals in Malaysia (confirmed on January 25, 2020) were still at large within society at that point in time. With a 4 days transmission rate, the next three individuals to be infectious would be on January 29, 2020, and the next six individuals to be infectious would be on February 2, 2020. On February 8, 2020, the first cohort of three individuals would display symptoms and be hospitalized. On February 12, 2020, the second cohort consisting of three individuals to be infectious on January 29, 2020, would display symptoms and be hospitalized. On February 16, 2020, the third cohort consisting of six individuals to be infectious February 2, 2020, will show symptoms and will be hospitalized. Prior to anybody from the first to the third cohort displaying symptoms and being hospitalized, the fourth cohort of twelve individuals to be infectious will occur on February 6, 2020. This cycle repeats itself ad infinitum until certain measures are able to halt the process. Figure 4 depicts this process.

We note here that the actual cumulative positive cases (where we assumed they are hospitalized and not in contact with other susceptible individuals) reported on March 29, 2020, is 2470 individuals. Meanwhile, the predicted cumulative number of infectious individuals to be hospitalized on March 29, 2020, is 3063. Furthermore, we have predicted that there are around 19,926 infectious individuals still roaming around in society on March 29, 2020.

Although the prediction depicted in Fig. 4 is of exponential growth of infectious individuals, we also take note of the following:

1. The above 'avalanche' effect is under the assumption that no remedial action has been taken to halt interaction (except for hospitalizing the infectious ones—which translates into no longer being in contact with susceptible individuals).
2. The above 'avalanche' effect is under the assumption that the best fit SIR curve upon the actual infectious count (discrete data) produces the transmission rate, $\beta$ = 0.22, that is translated into the idea that one infectious individual will transmit the disease to another individual within a 4-day interval.
3. On March 29, 2020, the MOH made an official announcement on the preparation of around 19,200 new beds^54^. This is a near coincidence to our prediction of 19,926 infectious individuals still roaming around within society on March 29, 2020.

Even though the SIR model is a numerical simulation, the numbers do provide us with a high possible scenario in which the COVID-19 infectious cases can surge too. This gives us an overall picture of the infectious severity of COVID-19 in Malaysia. As COVID-19 is still an infectious pandemic with some unclear properties, accurate SIR predictions can only be obtained once the outbreak has been successfully contained^16^. These trajectories could serve as a dependable means for the Malaysian government, businesses, and citizens to plan and mitigate for such spike in infectious cases. This study is believed to serve as one of the initial efforts for in-depth research on questions that revolve around this global pandemic within the Malaysian context. Our study is also a collective effort towards flattening the COVID-19 infectious curve in Malaysia and stopping the spread as per interventions set out by the government, namely the MCO. To our note, this early or preliminary study is part of ongoing wider research as more intensive studies regarding COVID-19 can be performed. An obvious future research direction for this study is to extend the current SIR model to the SEIR model by including the Exposed (*E*) compartment in the modelling procedure.

This study of modelling of data at the early stage of the pandemic in Malaysia is intended to provide useful insights for the planning of future outbreak control measures in the near future and is anticipated to be relevant for the coming years to curb the infectious rate. In line with Siam et al. and Johnson^55,56^, this study described the gaps found in public health preparedness, epidemiological characteristics, as well as mental preparedness of the Malaysian citizens to overcome this pandemic. The objectives of this study were to simulate the infectious trend and trajectory of Covid-19 in Malaysia and to determine the approximate number of days required for the trend to decline. The objectives were achieved by using the SIR model and data that were available at the time of writing.

However, the data used was collected over a period of only 110 days. Due to the limited availability of data and information at the time of writing, a standard SIR model was considered for this study. The major components considered in this study were limited to susceptible, infectious and recovered individuals. The exposed category was not considered here. Another limitation is that it did not take into account age dependent mixing pattern mainly due to the short term nature of this study and to understand the initial outbreak of the disease in a homogenous mixing setting. Control strategies were also not considered in this study as this was intended to simulate a disease outbreak without any interventions. This study can be extended further by including disease induced death in the context of an SEIR model. Furthermore, the effects of control strategies such as movement control order and vaccination can be incorporated into the model for analysis on the disease transmission dynamics.

## Methodology

### Data source

In this modelling study, we extracted the daily number of confirmed positive (infectious) cases from the official daily statistics of COVID-19 provided on the MOH web portal^57^. The extracted records were then collated as time-series data, which begins from day zero of the COVID-19 outbreak in Malaysia (January 25, 2020). In order to avoid any possibility of biases in using a single data source, we validated our figures with the Kini News Lab COVID-19 tracker^44^, a local website that provides real-time data and information on COVID-19. These data are collected through daily press conference statements by the Director-General of Health, Malaysia, where patients' data are not identifiable and remain anonymous. Hence, ethical approval is not required. Data were collected for the duration of only 110 days from day zero of the outbreak due to the limited data available at the time of writing. The first 67 days was chosen for simulation so as to complete a month from day zero until a month into the second wave, while the following 13 days (80 days in total) was used for the first projection for initial evaluation of the simulation and finally, the additional 30 days from first trajectory (total 110 days) was used for final evaluation of the simulation.

### Infectious trend simulation of COVID-19 in Malaysia

The population of Malaysia in 2020 is estimated at 32.7 million (mid-year estimation by the Department of Statistics, Malaysia), with a well-being index of 121 points (as of 2019), and as for living conditions, Malaysia ranked 55th out of 157 countries according to the World Bank's Human Capital Index. Being one of the famous tourist destinations in Southeast Asia and possessing good freedom of travel, Malaysia also attracts many tourists worldwide, with 25.8 million air passenger movements and 99,357 international flights in 2020^58^. Having taken into these figures into consideration, the modelling process is initiated.

**Figure 5:**
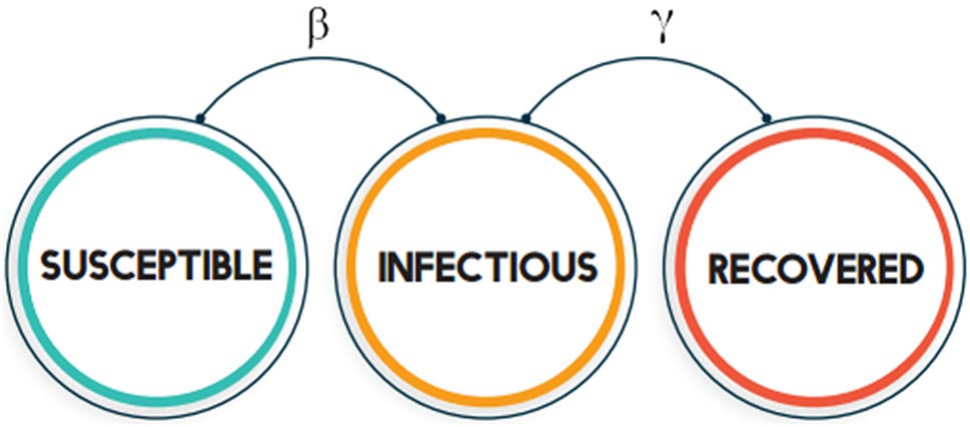
SIR state diagram.

In the modelling procedure, we divided the population into three compartments, as follows: susceptible ${\varvec{S}}\left({\varvec{t}}\right),$ number of not yet infectious and disease-free individuals at a time (${\varvec{t}}$), infectious ${\varvec{I}}\left({\varvec{t}}\right),$ the number of confirmed or isolated individuals at a time $({\varvec{t}}$), and recovered ${\varvec{R}}({\varvec{t}})$, no longer infectious individuals at a time (${\varvec{t}}$). We used the standard SIR epidemic model (Fig. 5) to simulate the infectious severity of COVID-19 in Malaysia beginning from the first day of the outbreak. This model is widely used as a first approach to analyze virus spreading and is reasonably predictive for infectious diseases which are transmitted from human to human, and where recovery confers lasting resistance, such as measles, mumps and rubella^59^.

The standard SIR model assumes no births or deaths, i.e. a fixed population, $N=S\left(t\right)+I\left(t\right)+R(t)$. The primary components of this model are the parameters $\beta$: transmission rate, which controls the rate of spread; and $\gamma$: recovery rate. If the average duration of recovery is denoted $D$, then the recovery rate is given by $\gamma =1/D$, since an individual experience one recovery in $D$ days. Individuals move from susceptible compartment to infectious compartment at the rate of $\beta$ and from infectious compartment to recovered compartment at the rate of $\gamma$.

Apart from these parameters, another important measure in epidemiology is the basic reproductive number ${R}_{0}$, which estimates the speed at which a disease is capable of spreading in a specific population^60^. The variable ${R}_{0}$ also indicates the number of secondary infections stemming directly from the first case in a susceptible population. When ${R}_{0}>1$, one infected individual will on average infect $>1$ person in total. When ${R}_{0}=1$, we are right at the threshold between an epidemic and not. Finally, when ${R}_{0}<1$, one infected individual will, on average, infect $<1$ person in total. Thus, it is the target to have mechanisms to achieve ${R}_{0}<1$. As disclosed by Tan Sri Dr Noor Hisham Abdullah, Director General of Health, Malaysia, on April 10 2020, Malaysia is approaching ${R}_{0}=1$^43^. This significant improvement is due to, among others—the Movement Control Order (MCO), better social distancing etiquettes and hygienic practices.

The dynamics of the COVID-19 transmission can be described using the following nonlinear ordinary differential equations (ODEs) as shown below:

$$\frac{dS}{dt}= -\frac{\beta SI}{N},$$

$$\frac{dI}{dt}= \frac{\beta SI}{N}- \gamma I,$$

$$\frac{dR}{dt}=\gamma I.$$

The differential equations were numerically solved with R software environment (version 3.6.3), with Runge–Kutta (RK4) method via the package deSolve (version 1.28)^42^. On the first day of the outbreak, there were three infectious cases reported with no recovered cases and to initialize each compartment, and we use $N=S\left(t\right)+I\left(t\right)+R(t)$, we obtain $S\left(0\right)=N-I\left(0\right)-R\left(0\right)$ which follows that $S\left(0\right)=\mathrm{32,731,000}-3-0=\mathrm{32,730,997}$ with $I\left(0\right)=3$ and $R\left(0\right)=0$. Although it may seem usual to work with the exact counts itself, the more apt way to obtain the initial conditions is by using fractional representation by dividing with $N$. This approach is reasonable for numerical simulations performed at the early stage of a pandemic since it evades discrepancies that may be caused by the large count of the population while having a significantly small counts in the infectious and recovered compartments^61^. Thus, $S(0)=S(0)/N=\mathrm{32,730,997}/\mathrm{32,731,000}\approx 0.999$, $R(0)=R(0)/N=0$, and hence $I\left(0\right)\approx 0.001$ in order to satisfy $N=S\left(0\right)+I\left(0\right)+R(0)$.

## References

[1] Coronavirus disease 2019 (COVID-19) Situation Report-94 HIGHLIGHTS.

[2] Verity R (2020). Estimates of the severity of coronavirus disease 2019: A model-based analysis. Lancet Infect. Dis..

[3] COVID-19 Public Health Emergency of International Concern (PHEIC) Global research and innovation forum. https://www.who.int/publications/m/item/covid-19-public-health-emergency-of-international-concern-(pheic)-global-research-and-innovation-forum. Accessed 12 Feb 2020.

[4] Statement on the second meeting of the International Health Regulations. Emergency Committee regarding the outbreak of novel coronavirus (2019-nCoV). (2005). https://www.who.int/news/item/30-01-2020-statement-on-the-second-meeting-of-the-international-health-regulations-(2005)-emergency-committee-regarding-the-outbreak-of-novel-coronavirus-(2019-ncov). Accessed 30 Jan 2020.

[5] WHO Coronavirus (COVID-19) Dashboard|WHO Coronavirus (COVID-19) Dashboard With Vaccination Data. https://covid19.who.int/. Accessed 5 Feb 2020.

[6] Coronavirus In Context. https://multimedia.scmp.com/widgets/china/viruscompare/. Accessed 6 May 2020.

[7] Advice for the public. https://www.who.int/emergencies/diseases/novel-coronavirus-2019/advice-for-public. Accessed 2 June 2020.

[8] Chan JFW (2020). A familial cluster of pneumonia associated with the 2019 novel coronavirus indicating person-to-person transmission: A study of a family cluster. Lancet.

[9] Chen D, Moulin B, Wu J (2015). Analyzing and Modeling Spatial and Temporal Dynamics of Infectious Diseases.

[10] Guan W (2020). Clinical characteristics of coronavirus disease 2019 in China. N. Engl. J. Med..

[11] Huang C (2020). Clinical features of patients infected with 2019 novel coronavirus in Wuhan, China. Lancet.

[12] Li Q (2020). Early transmission dynamics in Wuhan, China, of novel coronavirus-infected pneumonia. N. Engl. J. Med..

[13] Lauer SA, Grantz KH, Bi Q, Jones FK, Zheng Q, Meredith HR, Azman AS, Reich NG, Lessler J (2020). The incubation period of coronavirus disease 2019 (COVID-19) from publicly reported confirmed cases: Estimation and application. Ann. Intern. Med..

[14] Report of the WHO-China Joint Mission on Coronavirus Disease 2019 (COVID-19). https://www.who.int/publications/i/item/report-of-the-who-china-joint-mission-on-coronavirus-disease-2019-(covid-19). Accessed 28 Feb 2020.

[15] Symptoms of Coronavirus|CDC. https://www.cdc.gov/coronavirus/2019-ncov/symptoms-testing/symptoms.html. Accessed 22 Feb 2020.

[16] Malaysia announces movement control order after spike in Covid-19 cases (updated)|The Star. https://www.thestar.com.my/news/nation/2020/03/16/malaysia-announces-restricted-movement-measure-after-spike-in-covid-19-cases. Accessed 16 Mar 2020.

[17] Chen X, Yu B (2020). First two months of the 2019 Coronavirus Disease (COVID-19) epidemic in China: Real-time surveillance and evaluation with a second derivative model. Glob. Health Res. Policy.

[18] Kuhl E (2020). Data-driven modeling of COVID-19—Lessons learned. Extreme Mech. Lett..

[19] Poletto C, Scarpino SV, Volz EM (2020). Applications of predictive modelling early in the COVID-19 epidemic. Lancet Digit. Health.

[20] Kucharski AJ (2020). Early dynamics of transmission and control of COVID-19: A mathematical modelling study. Lancet Infect. Dis..

[21] Cooper BS, Pitman RJ, Edmunds WJ, Gay NJ (2006). Delaying the international spread of pandemic influenza. PLoS Med..

[22] Viboud C, Sun K, Gaffey R, Fumanelli L, Merler S, Zhang Q, Chowell G, Simonsen L, Vespignani A (2018). The RAPIDD Ebola forecasting challenge: Synthesis and lessons learnt. Epidemics.

[23] Aylward B, Barboza P, Bawo L, Bertherat E, Bilivogui P, Blake I, Brennan R, Briand S, Chakauya JM, Chitala K (2014). Ebola virus disease in West Africa—The first 9 months of the epidemic and forward projections. N. Engl. J. Med..

[24] Nishiura H, Klinkenberg D, Roberts M, Heesterbeek JAP (2009). Early epidemiological assessment of the virulence of emerging infectious diseases: A case study of an influenza pandemic. PLoS One.

[25] Chen D, Moulin B, Wu J (2015). Analyzing and Modeling Spatial and Temporal Dynamics of Infectious Diseases.

[26] Wangping J (2020). Extended SIR prediction of the epidemics trend of COVID-19 in Italy and compared with Hunan, China. Front. Med..

[27] Zahiri A, RafieeNasab S, Roohi E (2020). Prediction of peak and termination of novel coronavirus Covid-19 epidemic in Iran. medRxiv.

[28] Tang B (2020). Estimation of the transmission risk of the 2019-nCoV and its implication for public health interventions. J. Clin. Med..

[29] Wu JT, Leung K, Leung GM (2020). Nowcasting and forecasting the potential domestic and international spread of the 2019-nCoV outbreak originating in Wuhan, China: A modelling study. Lancet.

[30] Amira, F. Hamzah FA, Lau C, Nazri H, Ligot DV, Lee G, Tan CL, *et al.* CoronaTracker: Worldwide COVID-19 Outbreak Data Analysis and Prediction. [Preprint]. Bull World Health Organ. E-pub: 19 March 2020. 10.2471/BLT.20.25569.

[31] Yang Z, Zeng Z, Wang K, Wong SS, Liang W, Zanin M, Liu P, Cao X, Gao Z, Mai Z (2020). Modified SEIR and A.I. prediction of the epidemics trend of COVID-19 in China under public health interventions. J. Thorac. Dis..

[32] Musa SS, Qureshi S, Zhao S, Yusuf A, Mustapha UT, He D (2021). Mathematical modeling of COVID-19 epidemic with effect of awareness programs. Infect. Dis. Model..

[33] Asamoah JKK (2020). Global stability and cost-effectiveness analysis of COVID-19 considering the impact of the environment: Using data from Ghana. Chaos Solitons Fractals.

[34] Sun T, Weng D (2020). Estimating the effects of asymptomatic and imported patients on COVID-19 epidemic using mathematical modeling. J. Med. Virol..

[35] Ceylan Z (2020). Estimation of COVID-19 prevalence in Italy, Spain, and France. Sci. Total Environ..

[36] Giordano G (2020). Modelling the COVID-19 epidemic and implementation of population-wide interventions in Italy. Nat. Med..

[37] Asamoah JKK, Jin Z, Sun GQ, Seidu B, Yankson E, Abidemi A (2021). Sensitivity assessment and optimal economic evaluation of a new COVID-19 compartmental epidemic model with control interventions. Chaos Solitons Fractals.

[38] Yamamoto N, Jiang B, Wang H (2021). Quantifying compliance with COVID-19 mitigation policies in the US: A mathematical modeling study. Infect. Dis. Model..

[39] Wong, W. K., Juwono, F. H. & Chua, T. H. SIR simulation of COVID-19 pandemic in Malaysia: Will the vaccination program be effective? arXiv e-prints 2101.07494. https://arxiv.org/pdf/2101.07494.pdf (2021).

[40] Salman AM, Ahmed I, Mohd MH, Jamiluddin MS, Dheyab MA (2021). Scenario analysis of COVID-19 transmission dynamics in Malaysia with the possibility of reinfection and limited medical resources scenarios. Comput. Biol. Med..

[41] Abidemi A, Zainuddin ZM, Aziz NAB (2021). Impact of control interventions on COVID-19 population dynamics in Malaysia: A mathematical study. Eur. Phys. J. Plus.

[42] Soetaert K, Petzoldt T, Setzer RW (2010). Solving differential equations in R: Package deSolve. J. Stat. Softw..

[43] Ridenhour B, Kowalik JM, Shay DK (2018). Unraveling R0: Considerations for public health applications. Am. J. Public Health.

[44] Covid-19 in Malaysia|Kini News Lab. https://newslab.malaysiakini.com/covid-19/en. Accessed 4 Mar 2020.

[45] Terkini Harian|COVID-19 MALAYSIA. https://covid-19.moh.gov.my/terkini. Accessed 6 Mar 2020.

[46] Cruz MP, Santos E, Cervantes MAV, Juárez ML (2021). COVID-19, a worldwide public health emergency. Rev. Clin. Esp..

[47] del Rio C, Malani PN (2020). 2019 novel coronavirus—Important information for clinicians. JAMA.

[48] Han Y, Yang H (2020). The transmission and diagnosis of 2019 novel coronavirus infection disease (COVID-19): A Chinese perspective. J. Med. Virol..

[49] Walsh KA, Spillane S, Comber L, Cardwell K, Harrington P, Connell J (2020). The duration of infectiousness of individuals infected with SARS-CoV-2. J. Infect..

[50] Ahmed F, Ahmed N, Pissarides C, Stiglitz J (2020). Why inequality could spread COVID-19. Lancet Public Health.

[51] He X (2020). Temporal dynamics in viral shedding and transmissibility of COVID-19. Nat. Med..

[52] Lancet T (2020). India under COVID-19 lockdown. Lancet (London, England).

[53] Rozenfeld Y (2020). A model of disparities: Risk factors associated with COVID-19 infection. Int. J. Equity Health.

[54] Liu Y, Gayle AA, Wilder-Smith A, Rocklöv J (2020). The reproductive number of COVID-19 is higher compared to SARS coronavirus. J. Travel Med..

[55] Siam MHB (2021). Insights into the first seven-months of COVID-19 pandemic in Bangladesh: Lessons learned from a high-risk country. Heliyon.

[56] ERIC-EJ1260365-U.S. Faculty and Administrators’ Experiences and Approaches in the Early Weeks of the COVID-19 Pandemic, Online Learning. 2020. https://eric.ed.gov/?id=EJ1260365. Accessed 21 June 2020.

[57] Choi S, Ki M (2020). Estimating the reproductive number and the outbreak size of COVID-19 in Korea. Epidemiol. Health.

[58] Malaysia Airport Annual Report 2020. http://annualreport2020.malaysiaairports.com.my/. Accessed 31 Jan 2020.

[59] Chen, D., Bernard, M., Jianhong, W. Modeling the spread of infectious diseases: A review. In *Analyzing and Modeling Spatial and Temporal Dynamics of Infectious Diseases*. 19–42. 10.1002/9781118630013.ch2 (2014).

[60] Home|COVID-19 MALAYSIA. http://covid-19.moh.gov.my/. Accessed 6 Aug 2020.

[61] Smith, D., & Moore, L. The SIR model for spread of disease: the differential equation model. Loci. (2004).https://www.maa.org/press/periodicals/loci/joma/the-sir-model-for-spread-of-disease-the-differential-equation-model. Accessed 4 May 2020.

